# Case report: fatal bleeding from a duodenal ulcer—Dieulafoy’s lesion?

**DOI:** 10.1007/s00414-021-02721-w

**Published:** 2021-10-19

**Authors:** Julia Ulbricht, Burkhard Madea, Elke Doberentz

**Affiliations:** Institute of Legal Medicine, University Hospital Bonn, Stiftsplatz 12, 53111 Bonn, Germany

**Keywords:** Dieulafoy’s lesion, Large caliber vessel, Fatal hemorrhage, Gastrointestinal bleeding, Duodenal ulcer

## Abstract

A 46-year-old man was admitted to the hospital by ambulance due to syncope. A standard blood screening showed a normal Hb value. The man had known hemorrhoids and a single fresh rectal bleeding earlier at home. On the following morning, the patient suddenly required resuscitation within a few minutes and subsequently died. Autopsy revealed a fatal hemorrhage with blood loss in the stomach and small and large intestines and a mucosal defect of the duodenum. After autopsy, the question arose whether the cause of death might have been a rare Dieulafoy’s lesion—aim of this case report was to clarify the diagnosis.

## Introduction

Dieulafoy’s lesion, also called Dieulafoy’s ulcer or Dieulafoy’s disease, describes a large caliber arterial vessel that runs in the submucosa of the stomach, where usually smaller vessels are located. Because of the thin mucosal layer, due to a small mucosal defect, the artery can protrude into the lumen and is then exposed to the slightest mechanical irritation such as peristaltic shear stress. Disruption of the vessel can subsequently cause acute and severe (lethal) bleeding.

The lethality of acute gastrointestinal (GI) bleeding is 5–10%. About 85% of bleeding in the GI tract is upper gastrointestinal bleeding, which is more severe than bleeding in the lower GI tract. Erosions or ulcerations of the stomach or duodenum are the most frequent causes of GI bleeding, accounting for about 50% of the cases [1–3]. Dieulafoy’s lesion, which has a lethality of 8.6%, is a rare cause of upper gastrointestinal bleeding with an incidence of 1–2%. Dieulafoy’s lesion is difficult to diagnose and, therefore, likely accounts for at least some of the GI bleeding cases with unidentified bleeding sources [4–6].

Pathology and diagnostic criteria for Dieulafoy’s disease can be summarized as follows [4, 7–11]: The affected vessel has an abnormally wide caliber up to 1–3 mm diameter. There is a lack of mucosal inflammation, indicating no acid-peptide process. Histologically signs of deep ulceration or penetration of the muscularis mucosae are not present, and aneurysm, arteriosclerosis, or signs of vasculitis are generally absent.

Clinical presentation of Dieulafoy’s lesion includes asymptomatic patients without history of gastric complaints before showing symptoms of GI bleeding such as hematemesis, melena, or hematochezia. There is a higher incidence in older age (mean age 50 years), but it can occur at any age, and the male/female ratio is 2:1. The bleeding is often severe and painless, and the patients present with signs of hemodynamic instability, such as tachycardia or hypotension.

In the presented case, a 46-year-old man was hospitalized due to syncope. Pre-existing conditions were hemorrhoids and a previous rectal single bleeding. In the hospital, a standard blood test revealed no signs of blood loss, including a normal Hb value. The following morning, shortly after the breakfast, the patient suddenly collapsed and fell out of the bed. Initially, the patient was still responsive, but was not fully oriented. At that moment, he complained about shortness of breath and chest pain. The blood pressure at this time was only about 60 mmHg systolic with a normal heart rate of 60/min and a sinus rhythm. Within a few minutes, he required resuscitation and died.

## Autopsy findings

**Fig. 1:**
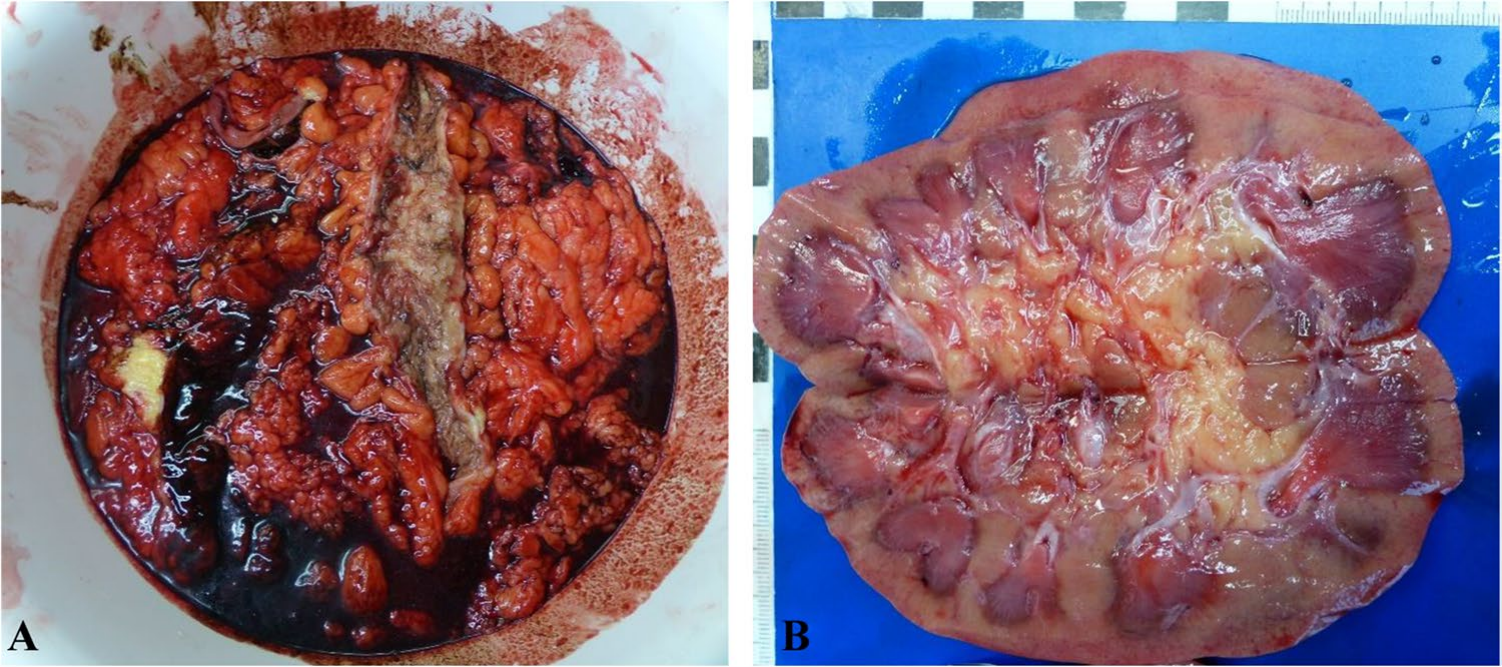
Autopsy findings showing blood suspect contents in the small and large intestines (**A**) and shock kidneys (**B**)

Autopsy revealed signs of high blood loss. More than 1 l of blood was found in the stomach and duodenum (1 l in the stomach, 120 ml in the duodenum). The entire small and large intestines were filled with blood suspect contents (Fig. 1a). Shock kidneys (Fig. 1b) and subendocardial hemorrhage of the left ventricle of the heart were present.

**Fig. 2:**
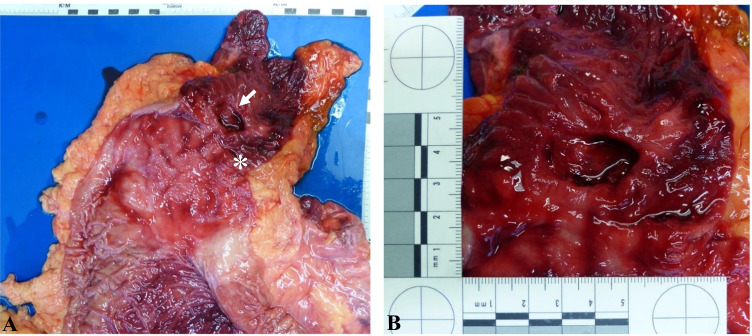
Autopsy findings revealing a mucosal defect in the duodenum ( ←) right below the pylorus (*)

A mucosal defect of about 3 cm in diameter and about 0.5 cm deep was found in the duodenum right below the pylorus (Fig. 2). No further mucosal defects were detected.

Other findings included a high heart weight (610 g), a fatty liver, and moderate arteriosclerosis.

On external examination, pale mucous membranes (conjunctivae, oral vestibular mucosae) were noted. There were no signs of external violence.

The cause of death was determined as internal hemorrhage from an ulcer of the duodenum. Subsequent to the autopsy, the question arose whether the ulcer might have been Dieulafoy’s disease; thus, histological examinations were performed.

## Histological findings

**Fig. 3:**
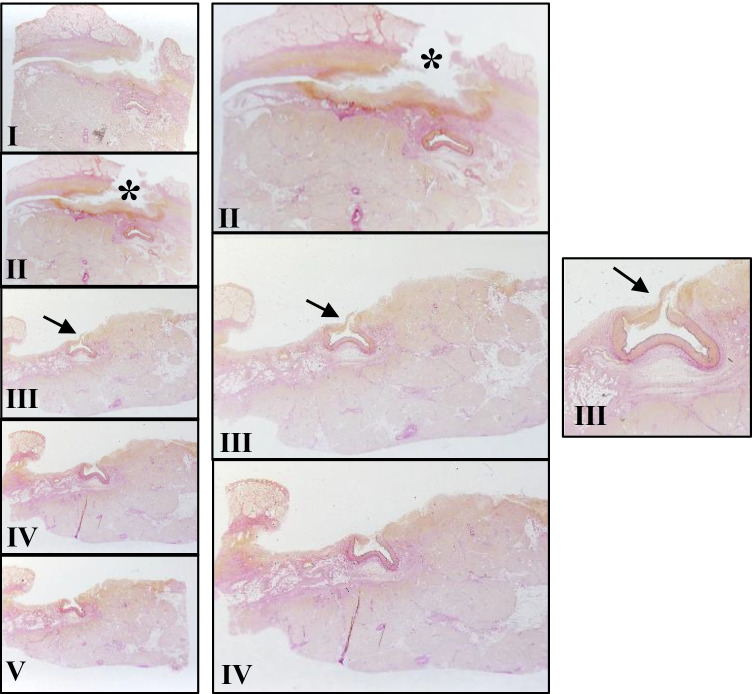
Extract of 5 slides (**I–V**) from the mucosal defect in the duodenum. The defect (*) in the duodenal stratification with underlying amorphous tissue is indicated. Slides III–V show loss of full thickness in the duodenal wall. The arrow ( ←) points to a disrupted arterial vessel in the duodenal lumen that caused the bleeding

**Fig. 4:**
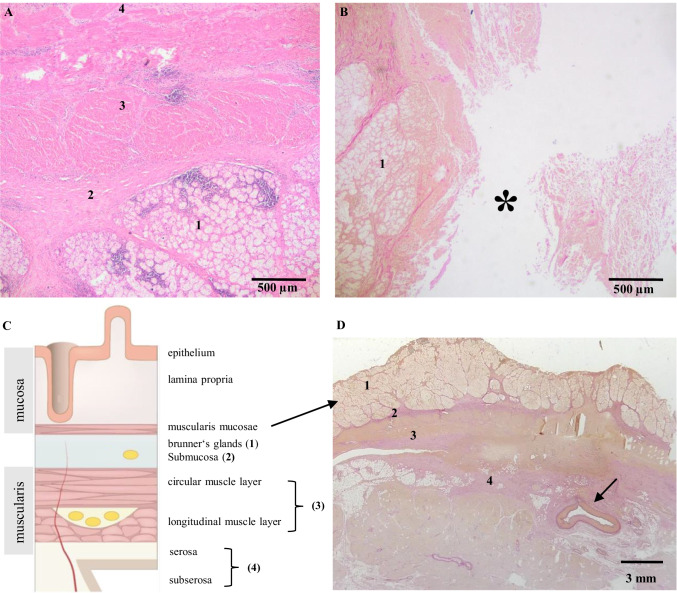
Preserved wall thickness (**A**) of the duodenum, hematoxylin and eosin staining, 20 × . **B** In the defect stratification is completely lost (see * from Fig. 3), Elastica van Gieson-Staining, 20 × . **C** Schematic view of duodenal layers (modified according to Amboss GmbH Berlin und Köln, Germany). **D** Preserved wall thickness containing later disrupted artery ( ←), picture of a slide

The mucosal defect of the duodenum was cut into 10 slices. Each slice was further cut into 15 thin sections and stained with hematoxylin and eosin (HE), Elastica van Gieson (EvG), and iron staining. The macroscopic examination of the slides already revealed a vessel that opened into the lumen of the duodenum and appeared to have large caliber (diameter of about 3.5 mm). In the surrounding tissue, the three layers of the duodenal mucosa were disrupted; this disruption was validated microscopically (Figs. 3 and 4).

**Fig. 5:**
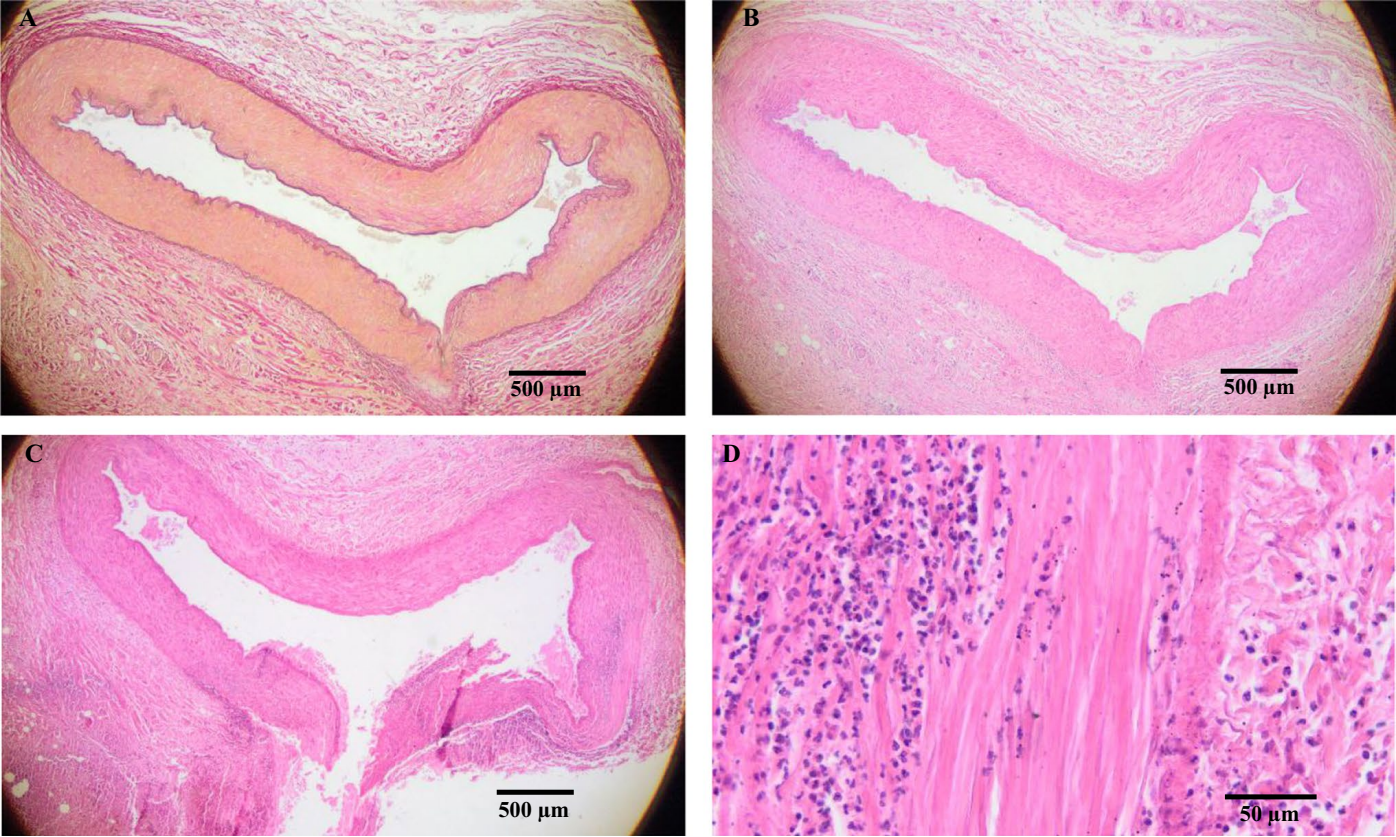
Intact artery with no pathologies shown in 20 × magnification using Elastica van Gieson (**A**) and hematoxylin and eosin staining (**B**). **C** Disrupted artery into the lumen of the duodenum. **D** At the site of the rupture, surrounding tissue and artery wall show acute reaction with inflammatory cells such as neutrophils, eosinophil granulocytes, and lymphocytes

Due to the numerous slices (150), the exact point at which the vessel was disrupted and caused the fatal bleeding could be determined. Microscopically the vessel could be identified as an arterial vessel, and no pathological vascular changes, such as vasculitis, aneurysm, or arteriosclerosis, were present. However, many inflammatory cells were found in the duodenal tissue at the site where the full thickness of the wall was lost (Fig. 5).

All iron stains were negative. A positive staining result requires survival for at least 3 days. Thus, the negative iron staining indicated fresh (acute bleeding) rather than bleeding that lasted few days, such as a seeping hemorrhage [12].

## Discussion

Some evidence supported the presence of Dieulafoy’s lesion in this case: An almost 50-year-old man suffered an acutely fatal upper GI bleeding with a single rectal bleeding the previous day. Shortly before his death, he presented with signs of hemodynamic instability such as low blood pressure and collapse. The Hb value, which was measured the previous day, was within the normal range. The histological examination revealed a conspicuous large caliber vessel (approximately 3 mm in diameter) disrupted to the lumen and caused the fatal hemorrhage. Also, no obvious abnormalities such as vasculitis or aneurysms were found in the affected artery.

However, the diagnosis of Dieulafoy’s lesion was not supported by the fact that histology revealed that the large caliber vessel did not run in the submucosa but in the serosa. The deeper the wall layer in the gastrointestinal tract, the larger caliber the vessels that run there. Because of a deep ulceration with inflammatory reaction, the full thickness of the duodenal mucosa in the surrounding tissue was completely lost and thus revealing an artery from deeper layers. Therefore, the artery that caused the fatal bleeding was not characteristic of Dieulafoy’s disease.

Macroscopically neither the vessel nor its defect was visible in the depth of the ulceration. Based on the anatomic localization, one might assume whether the disrupted vessel was a branch of the gastroduodenal artery. The ulceration was located in the duodenal mucosa right below the pylorus and immediately ventral to the pancreas, exactly where the gastroduodenal artery or it branches run. The gastroduodenal artery arises from the common hepatic artery of the coeliac trunk. It supplies blood directly to the pylorus and the proximal part of the duodenum and indirectly to the pancreatic head. Also, the gastroduodenal artery is known to cause fatal GI bleeding.

A thrombus in the vessel is sometimes observed after bleeding in Dieulafoy’s disease; but in this case, no thrombus was detected [13].

The duodenal mucosal defect was already visible at autopsy, but a precise evaluation of the stratification of the duodenal mucosa, the bleeding source, and, thus, the diagnosis of a Dieulafoy’s disease can only be determined with certainty using light microscopy.

This case demonstrates a GI bleeding due to deep ulceration leading to sudden lethal bleeding without prodromal symptoms, such as upper abdominal pain, use of nonsteroidal anti-inflammatory agents and alcohol in anamnesis, or signs of continuous blood loss in the GI tract. In this case, hemorrhoids probably caused the fresh single rectal bleeding.

The common term Dieulafoy’s ulcer is misleading and should possibly be avoided since some authors consider the “absence of deep ulceration” a requirement for the diagnosis of Dieulafoy’s disease [4, 9].

The case does not report something new or rare; the aim of this study was rather a didactic case report in which Dieulafoy’s disease is differentiated gradually from “normal” GI bleeding, helping to facilitate the diagnosis of this rare disease.

Taken into account the diagnostic criteria and pathology of Dieulafoy’s disease and in particular by performing histological examination, it was easily possible to exclude a Dieulafoy’s lesion in this case.
